# Supplementary material for: Probing plant signal processing optogenetically by two channelrhodopsins
Source: Nature. 2024 Aug 28;633(8031):872–7. doi: 10.1038/s41586-024-07884-1 (PMC11424491; doi:10.1038/s41586-024-07884-1)
Supplement: Supplementary file 1 — Coding sequences of XXM variants and legends for Supplementary Tables 1–8 and Videos 1 and 2. [file 41586_2024_7884_MOESM1_ESM.docx]

**Probing plant signal processing optogenetically by two channelrhodopsins**

Meiqi Ding^1#^, Yang Zhou^2#‡^, Dirk Becker^1#^, Shang Yang^2#^, Markus Krischke^3^, Sönke Scherzer^1^, Jing Yu-Strzelczyk^2^, Martin J. Mueller^3^, Rainer Hedrich^1*^, Georg Nagel^2*^, Shiqiang Gao^2*^, Kai R. Konrad^1*^

^1^ Molecular Plant Physiology and Biophysics, Julius-von-Sachs-Institute, University of Wuerzburg, 97082 Wuerzburg, Germany

^2^ Department of Neurophysiology, Physiological Institute, University of Wuerzburg, 97070 Wuerzburg, Germany

^3^ Pharmaceutical Biology, Julius-von-Sachs-Institute, University of Wuerzburg, 97082 Wuerzburg, Germany.

^‡^ current address: School of Life Sciences, Zhengzhou University, 450001, Zhengzhou, China

^#^these authors contributed equally.

*correspondence to: Kai R. Konrad, [kai.konrad@uni-wuerzburg.de](mailto:kai.konrad@uni-wuerzburg.de)

Shiqiang Gao, [gao.shiqiang@uni-wuerzburg.de](mailto:gao.shiqiang@uni-wuerzburg.de)

Georg Nagel, [nagel@uni-wuerzburg.de](mailto:nagel@uni-wuerzburg.de)

Rainer Hedrich, hedrich@botanik.uni-weurzburg.de

**Reporting Summary**

**Supplementary File 1. Coding sequences of XXM variants.**

**XXM 1.1 (5‘-3’): XXM(HQDH)-eYFP(the HQ and DH mutants are marked in red)**

ATGGATTATGGAGGCGCCCTGAGTGCCGTTGGGCGCGAGCTGCTATTTGTAACGAACCCAGTAGTCGTCAATGGCTCTGTACTTGTGCCTGAGGACCAGTGTTACTGCGCGGGCTGGATTGAGTCGCGTGGCACAAACGGTGCCCAAACGGCGTCGAACGTGCTGCAATGGCTTGCTGCTGGCTTCTCCATCCTACTGCTTATGTTTTACGCCTACCAAACATGGAAGTCAACCTGCGGCTGGGAGGAGATCTATGTGTGCGCTATCGAGATGGTCAAGGTGATTCTCGAGTTCTTCTTCGAGTTTAAGAACCCGTCCATGCTGTATCTAGCCACAGGCCACCGCGTCCAGTGGTTGCGTTACGCCGAGTGGCTTCTCACCTGCCCGGTCATTCTCATT**CAG**CTGTCAAACCTGACGGGCTTGTCCAACGACTACAGCAGGCGCACCATGGGTCTGCTTGTGTCT**CAT**ATTGGCACAATTGTGTGGGGCGCCACTTCCGCCATGGCCACCGGATACGTCAAGGTCATCTTCTTCTGCCTGGGTCTGTGTTATGGTGCTAACACGTTCTTTCACGCTGCCAAGGCCTACATCGAGGGTTACCACACCGTGCCGAAGGGCCGGTGTCGCCAGGTGGTGACTGGCATGGCTTGGCTCTTCTTCGTATCATGGGGTATGTTCCCCATCCTGTTCATCCTCGGCCCCGAGGGCTTCGGCGTCCTGAGCGTGTACGGCTCCACCGTCGGCCACACCATCATTGACCTGATGTCGAAGAACTGCTGGGGTCTGCTCGGCCACTACCTGCGCGTGCTGATCCACGAGCATATCCTCATCCACGGCGACATTCGCAAGACCACCAAATTGAACATTGGTGGCACTGAGATTGAGGTCGAGACGCTGGTGGAGGACGAGGCCGAGGCTGGCGCGGTACCCGCGGCCGCCACCATGGTGAGCAAGGGCGAGGAGCTGTTCACCGGGGTGGTGCCCATCCTGGTCGAGCTGGACGGCGACGTAAACGGCCACAAGTTCAGCGTGTCCGGCGAGGGCGAGGGCGATGCCACCTACGGCAAGCTGACCCTGAAGTTCATCTGCACCACCGGCAAGCTGCCCGTGCCCTGGCCCACCCTCGTGACCACCTTCGGCTACGGCCTGCAGTGCTTCGCCCGCTACCCCGACCACATGAAGCAGCACGACTTCTTCAAGTCCGCCATGCCCGAAGGCTACGTCCAGGAGCGCACCATCTTCTTCAAGGACGACGGCAACTACAAGACCCGCGCCGAGGTGAAGTTCGAGGGCGACACCCTGGTGAACCGCATCGAGCTGAAGGGCATCGACTTCAAGGAGGACGGCAACATCCTGGGGCACAAGCTGGAGTACAACTACAACAGCCACAACGTCTATATCATGGCCGACAAGCAGAAGAACGGCATCAAGGTGAACTTCAAGATCCGCCACAACATCGAGGACGGCAGCGTGCAGCTCGCCGACCACTACCAGCAGAACACCCCCATCGGCGACGGCCCCGTGCTGCTGCCCGACAACCACTACCTGAGCTACCAGTCCGCCCTGAGCAAAGACCCCAACGAGAAGCGCGATCACATGGTCCTGCTGGAGTTCGTGACCGCCGCCGGGATCACTCTCGGCATGGACGAGCTGTACAAG

**XXM 2.0 (5‘-3’): LR-XXM-11aa(HQDH)-T-eYFP-E (the HQ and DH mutants are marked in red)**

ATGCGACCCCAAATACTCCTCTTGCTGGCTTTGTTGACCCTTGGACTGGCTAACGGAACAGAAGGTCCCAACTTCTACGTTCCTTTCAGCAATAAGACAGGCGTAGTCAGATCCGGTAGTCGCGAGCTGCTATTTGTAACGAACCCAGTAGTCGTCAATGGCTCTGTACTTGTGCCTGAGGACCAGTGTTACTGCGCGGGCTGGATTGAGTCGCGTGGCACAAACGGTGCCCAAACGGCGTCGAACGTGCTGCAATGGCTTGCTGCTGGCTTCTCCATCCTACTGCTTATGTTTTACGCCTACCAAACATGGAAGTCAACCTGCGGCTGGGAGGAGATCTATGTGTGCGCTATCGAGATGGTCAAGGTGATTCTCGAGTTCTTCTTCGAGTTTAAGAACCCGTCCATGCTGTATCTAGCCACAGGCCACCGCGTCCAGTGGTTGCGTTACGCCGAGTGGCTTCTCACCTGCCCGGTCATTCTCATT**CAG**CTGTCAAACCTGACGGGCTTGTCCAACGACTACAGCAGGCGCACCATGGGTCTGCTTGTGTCT**CAT**ATTGGCACAATTGTGTGGGGCGCCACTTCCGCCATGGCCACCGGATACGTCAAGGTCATCTTCTTCTGCCTGGGTCTGTGTTATGGTGCTAACACGTTCTTTCACGCTGCCAAGGCCTACATCGAGGGTTACCACACCGTGCCGAAGGGCCGGTGTCGCCAGGTGGTGACTGGCATGGCTTGGCTCTTCTTCGTATCATGGGGTATGTTCCCCATCCTGTTCATCCTCGGCCCCGAGGGCTTCGGCGTCCTGAGCGTGTACGGCTCCACCGTCGGCCACACCATCATTGACCTGATGTCGAAGAACTGCTGGGGTCTGCTCGGCCACTACCTGCGCGTGCTGATCCACGAGCATATCCTCATCCACGGCGACATTCGCAAGACCACCAAATTGAACATTGGTGGCACTGAGATTGAGGTCGAGACGCTGGTGGAGGACGAGGCCGAGGCTGGCGCGGTAGTCGAGAAATCCAGAATTACTTCTGAAGGGGAGTATATCCCTCTGGATCAAATAGACATCAATGTTGTCGACACTAGTAGCAGAATGGTGAGCAAGGGCGAGGAGCTGTTCACCGGGGTGGTGCCCATCCTGGTCGAGCTGGACGGCGACGTAAACGGCCACAAGTTCAGCGTGTCCGGCGAGGGCGAGGGCGATGCCACCTACGGCAAGCTGACCCTGAAGTTCATCTGCACCACCGGCAAGCTGCCCGTGCCCTGGCCCACCCTCGTGACCACCTTCGGCTACGGCCTGCAGTGCTTCGCCCGCTACCCCGACCACATGAAGCAGCACGACTTCTTCAAGTCCGCCATGCCCGAAGGCTACGTCCAGGAGCGCACCATCTTCTTCAAGGACGACGGCAACTACAAGACCCGCGCCGAGGTGAAGTTCGAGGGCGACACCCTGGTGAACCGCATCGAGCTGAAGGGCATCGACTTCAAGGAGGACGGCAACATCCTGGGGCACAAGCTGGAGTACAACTACAACAGCCACAACGTCTATATCATGGCCGACAAGCAGAAGAACGGCATCAAGGTGAACTTCAAGATCCGCCACAACATCGAGGACGGCAGCGTGCAGCTCGCCGACCACTACCAGCAGAACACCCCCATCGGCGACGGCCCCGTGCTGCTGCCCGACAACCACTACCTGAGCTACCAGTCCGCCCTGAGCAAAGACCCCAACGAGAAGCGCGATCACATGGTCCTGCTGGAGTTCGTGACCGCCGCCGGGATCACTCTCGGCATGGACGAGCTGTACAAGTCTAGATTTTGCTATGAAAATGAAGTT

**Supplementary Table 1**

Exact numbers of samples in Fig. 3, Extended Data Fig. 7 and Extended Data Fig. 9.

**Supplementary Table 2**

Summary of DEGs and GO terms.

**Supplementary Table 3**

Functional classification of XXM specifically addressed genes.

**Supplementary Table 4**

Calcium related genes and sodium related genes triggered by XXM 2.0 stimulation.

**Supplementary Table 5**

Genes’ information involved in Fig. 4.

**Supplementary Table 6**

Green light addressed DEGs.

**Supplementary Table 7**

Genes’ information and primers for qPCR.

**Supplementary Table 8**

*P* values for significance analysis.

**Supplementary Video 1: Cytosolic free Ca^2+^ recording in *N. benthamiana* mesophyll cell in response to XXM 2.0 activation.**

Live-cell [Ca^2+^]_cyt_ imaging in the mesophyll of transiently transformed *N. benthamiana* leaves expressing Ret-XXM 2.0 upon green light (532 nm, 180 µW/mm^2^) illumination. Scale bar = 50 μm.

**Supplementary Video 2: Phenotypes of ACR1 2.0 or XXM 2.0 transgenic *N. tabacum* plants in red light when additional green light is switched on.**

Transgenic *N. tabacum* plants (Ret-eYFP, Ret-ACR1 2.0, Ret-XXM 2.0) were grown in red light (650 nm, 30 μW/mm^2^, 26 ℃) for 45 days and additional green light (520 nm, 9 μW/mm^2^) was added at t = 1 h. Only Ret-ACR1 2.0 *N. tabacum* leaves wilt at the edges after 4 hours of green light illumination, but when green light was switched off after 8 h illumination, turgor recovered within 20 min. Scale bar = 10 cm.
