# Supplementary material for: Probing plant signal processing optogenetically by two channelrhodopsins
Source: Nature. 2024 Aug 28;633(8031):872–7. doi: 10.1038/s41586-024-07884-1 (PMC11424491; doi:10.1038/s41586-024-07884-1)
Supplement: Supplementary file 2 — Reporting Summary [file 41586_2024_7884_MOESM2_ESM.pdf]

nature portfolio

Corresponding author(s): Kai R. Konrad, Shiqiang Gao, Rainer Hedrich and Georg Nagel

Last updated by author(s): Jul 9, 2024

Reporting Summary

Nature Portfolio wishes to improve the reproducibility of the work that we publish. This form provides structure for consistency and transparency in reporting. For further information on Nature Portfolio policies, see our Editorial Policies and the Editorial Policy Checklist.

Statistics

For all statistical analyses, confirm that the following items are present in the figure legend, table legend, main text, or Methods section.

n/a	Confirmed	
☐	☒	The exact sample size (*n*) for each experimental group/condition, given as a discrete number and unit of measurement
☐	☒	A statement on whether measurements were taken from distinct samples or whether the same sample was measured repeatedly
☐	☒	The statistical test(s) used AND whether they are one- or two-sided<br>*Only common tests should be described solely by name; describe more complex techniques in the Methods section.*
☐	☒	A description of all covariates tested
☐	☒	A description of any assumptions or corrections, such as tests of normality and adjustment for multiple comparisons
☐	☒	A full description of the statistical parameters including central tendency (e.g. means) or other basic estimates (e.g. regression coefficient) AND variation (e.g. standard deviation) or associated estimates of uncertainty (e.g. confidence intervals)
☐	☒	For null hypothesis testing, the test statistic (e.g. *F*, *t*, *r*) with confidence intervals, effect sizes, degrees of freedom and *P* value noted<br>*Give P values as exact values whenever suitable.*
☒	☐	For Bayesian analysis, information on the choice of priors and Markov chain Monte Carlo settings
☒	☐	For hierarchical and complex designs, identification of the appropriate level for tests and full reporting of outcomes
☒	☐	Estimates of effect sizes (e.g. Cohen's *d*, Pearson's *r*), indicating how they were calculated

Our web collection on statistics for biologists contains articles on many of the points above.

Software and code

Policy information about availability of computer code

Data collection	All data are collected with commercially available equipment and software. e.g. WinWCP V5.3.4 (University of Strathclyde, UK) for voltage or current recording; Leica LAS AF Version 2.7.3.9723 (Leica Microsystems, Wetzlar, Germany) for confocal images; VisiView Version 2.1.1 Software (Visitron Systems GmbH) for all-optical physiology experiments and Ca2+ imaging; The in vivo amperometric measurement of the production of H2O2 was performed by Patch-Master software V2x90 (HEKA) for ROS detection; Luminescence meaurements for ROS quantification were detected by Skanlt software Version 6.1; Analyst version 1.6.3 and MultiQuant Version 3.0.2 software from Sciex were used for mass spec detection of hormones and metabolites; Aequorin luminescence was detected by a photomultiplier (Photo Counting Module MP 1983 RS CPM, Perkin Elmer) controlled by IGI-MPRS232 (IGIsystems) and Labview V14.0.0 (National Instruments) were used in this measurement; Proline content was quantified by a spectrophotometer (lab/ Hitachi U-1500); Eppendorf Mastercycler ep realplex Version 2.2 software for qRT-PCR measurements; Quantification of photosynthetic performance was done with a Maxi PAM fluorometer controlled by the IMAGING WIN v2.41a FW MULTI RGB software (Walz, Effeltrich, Germany); The ChR2 structure model was generated with the standard molecular viewer PyMOL and the internal cavities were calculated using Hollow suit, which is a published Python script; In the framework of the transcriptomic analysis, transcript mapping to the tobacco genome was peformed with the Amalgkit software pipeline (https://github.com/kfuku52/amalgkit), for gene annotation the Dicots PLAZA 5.0 repository was used; Differential gene expression analysis and subsequent gene ontology analysis and heat map presentations was performed with the DIANE package, Venn diagrams were generated with the GOVenn script of the GOPlot package, GO analysis on Venn subsets was carried out with the help of gprofiler2, all R-packages used are described in detail within the Materials and Methods section.

Data analysis	Data analysis are all processed by commercially available software. e.g. ImageJ-win 64/FIJI for image analysis; Videos were generated by Adobe Premiere Pro CC 2015 with H.264 encoding. Origin Pro 2019 and GraphPad Prism v8.4.2 for graphs ; Excel (Office Professional) for source data handling; Student's t-test or One-way analysis of Variance (ANOVA) were used to analyze significance using IBM SPSS statistics (version 26.0).

For manuscripts utilizing custom algorithms or software that are central to the research but not yet described in published literature, software must be made available to editors and reviewers. We strongly encourage code deposition in a community repository (e.g. GitHub). See the Nature Portfolio guidelines for submitting code & software for further information.

Data

Policy information about availability of data

All manuscripts must include a data availability statement. This statement should provide the following information, where applicable:

- Accession codes, unique identifiers, or web links for publicly available datasets
- A description of any restrictions on data availability
- For clinical datasets or third party data, please ensure that the statement adheres to our policy

All the data generated in this study are available in the paper and the Supplementary Information. Source data behind graphs are provided with this paper. The RNA-seq data have been deposited in the National Center for Biotechnology Information (NCBI) database (Bioproject ID: PRJNA1108451).

Research involving human participants, their data, or biological material

Policy information about studies with human participants or human data. See also policy information about sex, gender (identity/presentation), and sexual orientation and race, ethnicity and racism.

Reporting on sex and gender	NA
Reporting on race, ethnicity, or other socially relevant groupings	NA
Population characteristics	NA
Recruitment	NA
Ethics oversight	NA

Note that full information on the approval of the study protocol must also be provided in the manuscript.

Field-specific reporting

Please select the one below that is the best fit for your research. If you are not sure, read the appropriate sections before making your selection.

☒ Life sciences ☐ Behavioural & social sciences ☐ Ecological, evolutionary & environmental sciences

For a reference copy of the document with all sections, see nature.com/documents/nr-reporting-summary-flat.pdf

Life sciences study design

All studies must disclose on these points even when the disclosure is negative.

Sample size	No sample sizes were predetermined by an statistical method. The sizes of samples were determined depended on experimental trials to allow for confident statistical tests. For each experiment, in Xenopus laevis oocytes we have at least 3 biological samples. For plants experiments, we included 2 transgene lines and more than 4-5 biological repeats for each. For transcriptomics analysis, 3 biological replicates were used. At least two times of biological repetition were done for each experiment.
Data exclusions	We excluded some electrical measurement due to the losing of electrical signals (the electrodes were not in the cell anymore or the cells were dead) during measurement. We also excluded some Ca2+ or pH measurement due to the losing of focus of cells during measurement. For the images of leaves, plants and oocytes, we have made many biological repeats with similar results, but did not show them all due to space limitations and the sake of clarity.
Replication	All the experimental replications were very successful and clear. The numbers of biological replicates were performed with more than 2 independent batches of plants or Xenopus laevis oocytes. The repeat numbers are listed in the legend part.
Randomization	For global green light treatment, all tobacco plants of different genotypes were marked on pots and randomly placed in the light controlled growth chamber. In order to verify the reliability and reproducibility of results, we always choose the materials in experiments randomly. If allocation was needed, the grouped samples were randomly allocated for each experiment. For instance, to detect all trans retinal production in the transgene tobacco lines, we took 10 leaves of different plants randomly from each line for the detection. In the metabolites measurement or transcriptome analysis, we also collected the same age leaves randomly from independent plants of different lines. For

Xenopus laevis oocytes experiments, we picked out the healthy oocytes first and separated them randomly for each expression vectors. We always use oocytes from the same supplier in one comparison experiment.

Blinding

In this study, there was no blinding test, because the differences among different experimental groups were extremely obvious. In addition to that all the experiment showed very good replication with enough biological repeats.

Reporting for specific materials, systems and methods

We require information from authors about some types of materials, experimental systems and methods used in many studies. Here, indicate whether each material, system or method listed is relevant to your study. If you are not sure if a list item applies to your research, read the appropriate section before selecting a response.

Materials & experimental systems

n/a	Involved in the study
☒	☐ Antibodies
☒	☐ Eukaryotic cell lines
☒	☐ Palaeontology and archaeology
☐	☒ Animals and other organisms
☒	☐ Clinical data
☒	☐ Dual use research of concern
☒	☐ Plants

Methods

n/a	Involved in the study
☒	☐ ChIP-seq
☒	☐ Flow cytometry
☒	☐ MRI-based neuroimaging

Animals and other research organisms

Policy information about studies involving animals; ARRIVE guidelines recommended for reporting animal research, and Sex and Gender in Research

Laboratory animals

Oocytes of Xenopus laevis frogs bought from EcoCyte Bioscience (Dortmund Germany) or obtained from frogs in the Julius-von-Sachs-Institute.

Wild animals

The study did not involve wild animals.

Reporting on sex

This information has not been collected. We use oocyte cells and sex was not considered in study design.

Field-collected samples

The study did not involve samples from the field.

Ethics oversight

Xenopus laevis oocytes were either bought from EcoCyte Bioscience (Dortmund Germany) or obtained from frogs in the Julius-von-Sachs-Institute, Würzburg University. The laparotomy to obtain oocytes from Xenopus laevis was carried out in accordance with the principles of the Basel Declaration and recommendations of Landratsamt Wuerzburg Veterinaeramt. The protocol under License #70/14 from Landratsamt Wuerzburg, Veterinaeramt, was approved by the responsible veterinarian.

Note that full information on the approval of the study protocol must also be provided in the manuscript.

Plants

Seed stocks

N. tabacum transgeic lines were generated in Molecular Plant Physiology and Biophysics, Julius-von-Sachs-Institute, University of Wuerzburg, 97082 Wuerzburg, Germany. UBQ10_Ret_ACR1 2.0 #1 and #2 , UBQ10_Ret_XXM 1.2 #1 and #2, UBQ10_Ret_XXM 2.0 #1 and #2, UBQ10_Ret_eYFP #1 and #2, UBQ10_ Ret-XXM 2.0/35s_NES_2*R-GECO1 #1, UBQ10_ Ret-XXM 2.0/35s_NES_2*pHuji #1.

Novel plant genotypes

N. tabacum transgeic lines were generated by bath the leaf discs in MS medium containing A. tumefaciens strain GV3101 harboring the pCAMBIA3300 vector with BASTA resistance or pCAMBIA1300 vector with hygromycin resistance. Explants and calli were generated on the Medium and the explants were moves to rooting medium. Finally got the trangene plants and seeds. At least 2 independent lines were selected to verify the genotypes.

Authentication

The transgenic plants were verified by eYFP, pHuji or R-GECO1 fluorescence in leaves. Seeds of individual plant were collected and selected for BASTA or hygromycin resistance using selection medium. PCR using gDNA as the template was applied to confirm the transgenic lines. The growth phenotype, biomass, photosynthesis parameters and the leaf carotenoid were compared between the transgene lines and wild type plants to check the influence.
